# Childhood pneumonia diagnostics: community health workers’ and national stakeholders’ differing perspectives of new and existing aids

**DOI:** 10.1080/16549716.2017.1290340

**Published:** 2017-05-09

**Authors:** Hollie Spence, Kevin Baker, Alexandra Wharton-Smith, Akasiima Mucunguzi, Lena Matata, Tedila Habte, Diana Nanyumba, Anteneh Sebsibe, Thol Thany, Karin Källander

**Affiliations:** ^a^ Department of Public Health Sciences, Karolinska Institutet, Stockholm, Sweden; ^b^ Malaria Consortium, London, UK; ^c^ Malaria Consortium, Phnom Penh, Cambodia; ^d^ Malaria Consortium, Kampala, Uganda; ^e^ Malaria Consortium, Juba, South Sudan; ^f^ Malaria Consortium, Addis Ababa, Ethiopia

**Keywords:** Childhood pneumonia, low-income country, diagnostic tools, pulse oximeter, respiratory rate counting

## Abstract

**Background:** Pneumonia heavily contributes to global under-five mortality. Many countries use community case management to detect and treat childhood pneumonia. Community health workers (CHWs) have limited tools to help them assess signs of pneumonia. New respiratory rate (RR) counting devices and pulse oximeters are being considered for this purpose.

**Objective:** To explore perspectives of CHWs and national stakeholders regarding the potential usability and scalability of seven devices to aid community assessment of pneumonia signs.

**Design:** Pile sorting was conducted to rate the usability and scalability of 7 different RR counting aids and pulse oximeters amongst 16 groups of participants. Following each pile-sorting session, a focus group discussion (FGD) explored participants’ sorting rationale. Purposive sampling was used to select CHWs and national stakeholders with experience in childhood pneumonia and integrated community case management (iCCM) in Cambodia, Ethiopia, Uganda and South Sudan. Pile-sorting data were aggregated for countries and participant groups. FGDs were audio recorded and transcribed verbatim. Translated FGDs transcripts were coded in NVivo 10 and analysed using thematic content analysis. Comparative analysis was performed between countries and groups to identify thematic patterns.

**Results:** CHWs and national stakeholders across the four countries perceived the acute respiratory infection (ARI) timer and fingertip pulse oximeter as highly scalable and easy for CHWs to use. National stakeholders were less receptive to new technologies. CHWs placed greater priority on device acceptability to caregivers and children. Both groups felt that heavy reliance on electricity reduced potential scalability and usability in rural areas. Device simplicity, affordability and sustainability were universally valued.

**Conclusions:** CHWs and national stakeholders prioritise different device characteristics according to their specific focus of work. The views of all relevant stakeholders, including health workers, policy makers, children and parents, should be considered in future policy decisions, research and development regarding suitable pneumonia diagnostic aids for community use.

## Background

Globally, pneumonia is one of the leading causes of death amongst children under five years of age. It is estimated that 15% of all child deaths can be attributed to pneumonia [1,2]. The vast majority of the 120 million annual cases of childhood pneumonia occur in low- and middle-income countries (LMIC), particularly those in sub-Saharan Africa and Southeast Asia [3].

The World Health Organization (WHO) defines childhood pneumonia as the presence of cough and/or difficult breathing with an elevated respiratory rate (RR) [4]. This classification is used in the WHO Integrated Management of Childhood Illness (IMCI) and integrated community case management (iCCM) guidelines to enable presumptive diagnosis of pneumonia according to clinical features. For children presenting with cough and/or difficult breathing, this approach requires counting the child’s RR for one minute, usually with the assistance of the United Nations Children’s Emergency Fund (UNICEF)-issued acute respiratory infection (ARI) timer [5] to track the necessary counting time. The accuracy of this method of counting is low amongst community health workers (CHWs) [6–8], contributing to issues of both overtreatment and under-treatment amongst children with pneumonia [9].

Recently, there has been interest in utilising new technology to create simple tools that assist frontline health workers in identifying and classifying signs of childhood pneumonia, through RR counting and other novel methods [10]. Pulse oximeters can detect hypoxemia (SpO_2_ < 90%), a clinical feature of severe pneumonia warranting oxygen therapy [11]. As well as indicating severe disease, hypoxemia in children with pneumonia is a significant risk factor for mortality [12–14]. Measuring children’s oxygen saturation in community settings could improve identification of severe disease requiring oxygen therapy and predict cases where oral antibiotic treatment may fail [15,16].

In medical settings, RR counting devices and pulse oximeters are not referred to as ‘diagnostic’ tools for pneumonia, because altered RRs and oxygen saturations occur in many diseases. However, an elevated RR in children with cough is predictive of pneumonia and is used in the iCCM/IMCI context by frontline health workers to identify suspected pneumonia. If pulse oximetry is incorporated into future pneumonia guidelines, hypoxemia will likely be a diagnostic indicator of severe disease requiring referral to a health facility with oxygen. For these contextual reasons the devices in this study are referred to as pneumonia diagnostic aids.

To achieve maximum impact, the use of new diagnostic aids should be prioritised by Ministries of Health (MoH) and integrated into national guidelines [17]. There is a lack of data on the views of end users (frontline health workers) and procurers, such as MoH and non-governmental organisation (NGO) staff, which could ideally inform the development of diagnostic aids. Moreover, there is limited knowledge of CHWs’ perceptions of which characteristics are important for pneumonia diagnostic aids. This study sought to gain insights from two principal groups of stakeholders, CHWs and national stakeholders, in four countries located in Southeast Asia and sub-Saharan Africa; Cambodia, Ethiopia, Uganda and South Sudan.

## Methods

### Study countries

All four study countries have high under age-five mortality rates with a significant burden of childhood pneumonia (Table 1). The MoH in each country sanctions programmes using CHWs to diagnose and treat children under five years with pneumonia through the framework of iCCM [18]. CHWs are mainly situated in rural, isolated and resource-poor areas of countries with limited access to formal medical care. The selection of countries was based on their relevant iCCM operating context, Malaria Consortium’s capacity in each country and to provide a meaningful study incorporating a broad range of contextual settings in the final results. All countries selected have a high proportion of under-five death caused by pneumonia (16–21%) and all are implementing MoH-defined policies where pneumonia is assessed and treated at community level. However, the CHW program delivering these services varies substantially in how it is set up in the different countries, in terms of length of training, literacy level of the CHWs and RR timing devices used by CHWs (Table 2).

**Table 1.** Under-five mortality and pneumonia statistics for each study country.

	Under-five mortality rate 2013 (per 1000 live births)[19]	Percentage of under-five mortality caused by pneumonia (%)[20]	Children with pneumonia symptoms who receive antibiotics 2009–2013 (%)[19]
Cambodia	38	17	39
Ethiopia	64	18	7
South Sudan	99	20	33
Uganda	66	16	47

**Table 2.** Overview of community health worker (CHW) programs in the study countries.

	Cambodia	Ethiopia	South Sudan	Uganda
Number of CHWs	800	27,000	8000	30,000
Local name for CHW	Extended village malaria worker (eVMW)	Health extension worker (HEW)	Community drug distributor (CDD)	Village health team member (VHT)
Length of initial training	5 days (2 days’ malaria training and 3 days’ sick child case management training)	1 year	6 days	11 days (5 days’ basic training and 6 days’ sick child management training)
Literacy level	Low	High	Extremely low	Low–medium
Current pneumonia diagnostic tool	ARI timer	Wrist watch/ARI timer	ARI timer with beads	ARI timer
Catchment area	130–150 households	400–500 households	250–300 households	250–500 households
Average case load per month	8	12	9	12

CHWs in each country are referred to by different local names: extended village malaria workers (Cambodia); health extension workers (Ethiopia); community drug distributors (South Sudan); and village health team members (Uganda). For the sake of simplicity, the term ‘community health workers’ was used to collectively refer to these groups.

### Study design

Qualitative methodology was used in this study. The combination of pile-sorting activities and focus group discussions (FGDs) was designed to comprehensively explore participant attitudes and perspectives towards various diagnostic devices.

### Sampling and participants

Sampling of all participants was purposive. Invitations to attend were extended to a large range of regional and national programme implementers involved in child health and pneumonia. This included representatives of national and regional MoH, regional health bureaus, multilateral organisations such as UNICEF, and relevant NGO staff working at regional and national levels. CHW participants were selected from lists of active CHWs in the relevant country region: Ratanakiri province in Cambodia; Southern Nations, Nationalities and People’s Regional State in Ethiopia; Mpigi district in the Central Region of Uganda; and Aweil centre and west counties in Northern Bahr El Ghazal state in South Sudan. Practical experience in diagnosing children with pneumonia was required.

**Figure 1.**
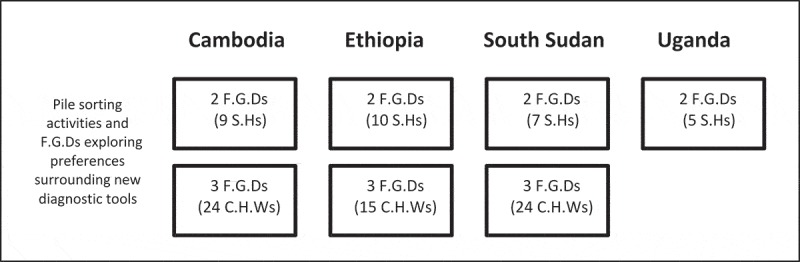
Number of FGDs held in each study country with total participant numbers in brackets. Notes: SH = stakeholder, CHW = community health worker.

Pile-sorting activities with accompanying FGDs were held with national stakeholders in each country. All countries held an additional three FGDs with CHWs, with the exception of Uganda due to time constraints (Figure 1). A total of 31 national and regional stakeholders and 63 CHWs participated. The numbers of FGDs were chosen to achieve maximum thematic saturation amongst both groups of participants, balanced with the available time and resources.

### Data collection

FGDs were held with national stakeholders and CHWs, in conjunction with a pile-sorting exercise of pneumonia diagnostic devices during December 2014 and January 2015 across the four countries. Pile sorting is a qualitative method used mainly in social sciences and health research. It aims to capture participants‘ opinions or experiences by having them sort word, item or picture cards into piles that classify a range of opinions or categories of interest [21]. Conducting FGDs immediately following the pile-sorting activity was designed to capture and explore participants’ decision-making rationale for their sorting.

Seven device types were demonstrated to participants with an accompanying explanation of their functions and costs (Table 3). Following the demonstration, devices were handed around amongst participants and a question and answer session was held.

**Table 3.** Descriptions of device types used in the pile-sorting activity.

Device type	Device description	Example device	Features	Price
Non-automated	Non-automated devices include tools that support the manual counting of chest movements, by indicating when to start and stop counting. Currently produced by Moneray and procured by UNICEF	Acute Respiratory Infection (ARI) timer	Durable; low cost; long battery life; easy to use; requires little training	USD 4.82
Assisted counting RR device	Devices that support an assisted count, by automating or negating the need for manual counting of each chest movement. Mobile software applications in the category of assisted count work when CHWs tap the screen or press buttons for each chest movement. Can run on feature phones or simple smartphones	RR smartphone app	Easy to use; fast result; doesn’t require counting skills; gives outcome	Free + cost of phone (approx. 70–150 USD)
		Respirometer Feature phone app	Easy to use; fast result; doesn’t require counting skills; low cost; gives outcome	Free + cost of phone (approx. 20–50 USD)
Handheld pulse oximeter	Oximeters that were traditionally designed for professional rather than for home use. Many of them are suitable for adults, children and young infants, since oxygen is measured using a finger external sensor that can be purchased separately for paediatric and neonatal use	Lifebox	Rechargeable; long warranty life; robust; reusable probes	USD 250
Mobile phone pulse oximeter	These devices function by connecting a pulse oximeter to a smartphone and then reading the oxygen saturation of the patient using an app installed on the phone with the oxygen values of the patient shown on the display of the phone. Requires a more advanced Android smartphone	Masimo iSpO2	Anti-motion technology; neonatal probe; high-spec phone required	USD 150 + cost of phone (approx. 150–200 USD)
Finger-tip pulse oximeter	These affordable, easy-to-use pulse oximeter devices incorporate the probe and the device in one unit	Contec	Rechargeable batteries (charger provided)	USD 40

These included:

- The ARI timer (and additional counting beads in South Sudan), the current tool used for pneumonia diagnosis amongst CHWs in all four countries;
- A fingertip pulse oximeter;
- A handheld pulse oximeter;
- A smartphone application which tracks the breaths counted when the screen is tapped on;
- A simple feature phone application which tracks the breaths counted when a button is pressed;
- A pulse oximeter probe attaching to a smartphone;
- A joint device combining the RR counting application and a pulse oximeter probe using the same smartphone (only shown to some groups in Ethiopia, South Sudan and Uganda).

Individual participants placed cards with various device names into different piles according to their perceived usability, and again for their perceived scalability. The piles were pre-determined by the researchers (Table 4). The facilitator recorded the overall pile-sorting results (i.e. how many times a device was placed into each pile) in a pro forma results table.

**Table 4.** Definitions of stage two pile-sorting categories.

Usability	
Pile 1	Able to use the device in the community setting
Pile 2	Able to use the device with reservations in the community setting
Pile 3	Possibly unable to use the device in the community setting
Pile 4	Unable to use the device in the community setting
**Scalability**	
Pile 1	Feasible to scale up
Pile 2	Feasible with reservations to scale up
Pile 3	Possibly unfeasible to scale up
Pile 4	Unfeasible to scale up

During the FGD, participants were questioned on the rationale behind their sorting choices using a uniform semi-structured question guide. This guided the discussion and ensured the same points were covered for each device, including the positive and negative aspects of the devices which participants felt were relevant to usability and scalability. Trained facilitators moderated the activities and FGDs in the participants’ local languages, or in English if participants were fluent. Participant numbers determined whether debriefing was conducted in FGD or interview format. FGDs/interviews were conducted in private meeting rooms and were audio recorded in full. Verbatim transcriptions (and translations if required) were performed afterwards. Full transcripts were used for analysis purposes. Research assistants recorded field notes throughout each FGD. Afterwards these were summarised into ‘fairnotes’ and were used for the preliminary analysis of results in each country [22].

In Cambodia, the full transcript for one national stakeholder FGD was not available and instead only the fairnotes were used for analysis purposes. In Uganda, two national stakeholders declined to participate in the pile-sorting activity or interviews but instead described their opinions on the devices in written form. Despite not conforming to the research protocol, this data was included in the analysis as the main interview questions were addressed.

### Data analysis

Results from the pile-sorting exercises were collated in Microsoft *Excel* (2011). All transcripts were imported into NVivo 10 software [23] for sorting and coding. Codes were assigned using a combination of inductive and deductive approaches. These codes and sub-categories were categorised and thematic content analysis [24] was performed.

Comparative interpretation [25] was performed on the data to explore the differences and similarities in thematic content between countries and between CHWs, national and regional stakeholders. This method allowed exploration into which opinions and experiences were universal, compared to those that were country or group specific, and the possible explanations for these phenomena.

## Results

### Pile-sorting activity

**Figure 2.**
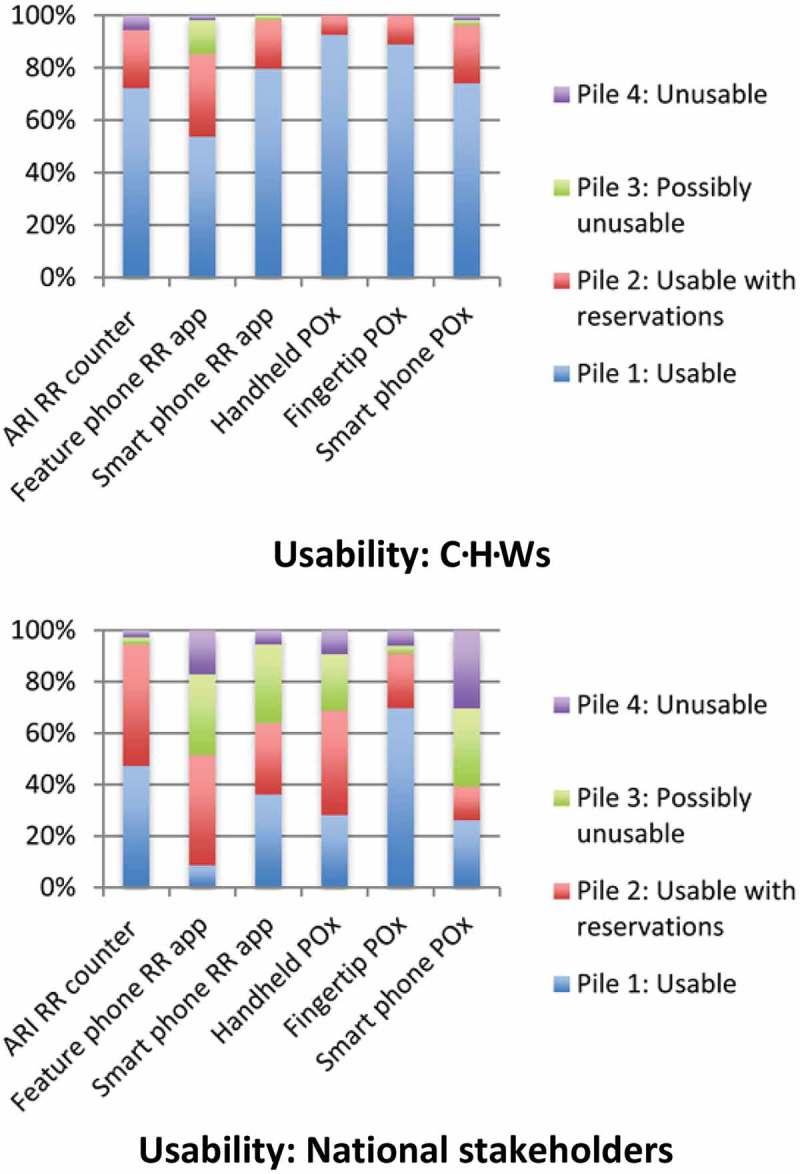
Graphs depicting the pile-sorting allocations of community health workers (CHWs) and national stakeholders in relation to usability (joint device not included due to low participant numbers).

**Figure 3.**
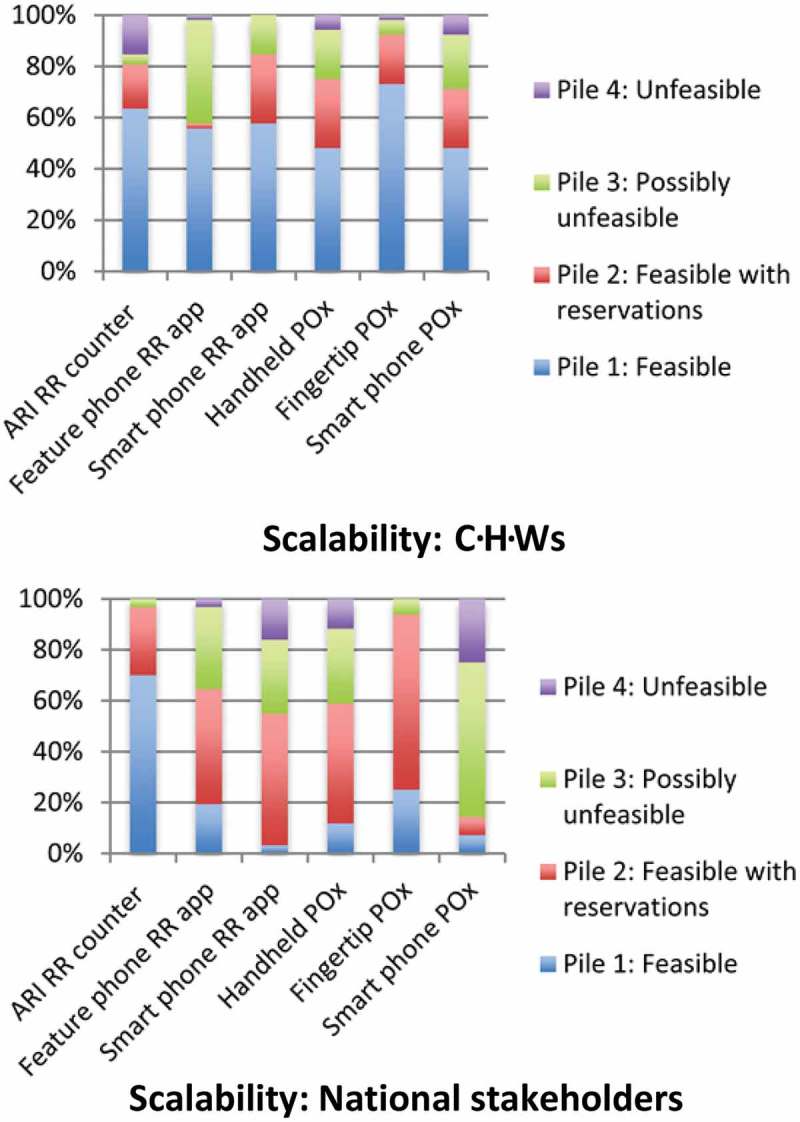
Graphs depicting the pile-sorting allocations of community health workers (CHWs) and national stakeholders in relation to scalability (joint device not included due to low participant numbers).

The full results of the pile-sorting activity can be seen in Figures 2 and 3. Overall, a much higher percentage of CHWs placed devices in pile 1 (able to use or scale up, with or without reservations) compared with national stakeholders. Both groups rated the fingertip pulse oximeter and the device currently used in standard practice, the ARI timer, highly in regards to usability. National stakeholders were extremely positive about the scalability potential of the ARI timer compared to the other device options (70% placed it in pile 1 and none in pile 4). CHWs had more varied perceptions of this device’s scalability (63% and 15% placed it in groups 1 and 4, respectively). National stakeholders rated the scalability and usability of the smartphone pulse oximeter poorly (86% placed it in pile 3 or 4 for scalability and 61% did so for usability). The fingertip pulse oximeter’s usability and scalability were rated highly by both national stakeholders and CHWs.

### Themes arising from FGDs

## Cost

CHWs and national stakeholders in all countries deemed the price of pneumonia diagnostic aids to be a significant factor in determining future scale-up. Many national stakeholders questioned who would be responsible for supplying the aids to large numbers of CHWs in each country. They generally expressed a preference for less expensive devices, such as the ARI timer and the fingertip pulse oximeter, and this is reflected in their scalability pile-sorting results (see Figure 3). CHWs also raised this issue and suggested if a device had a high price, donors would be unlikely to fund their purchase. Other CHWs were concerned they may be required to buy their own device, or pay for broken devices and stated they could not afford the high-cost items such as smartphones, the smartphone pulse oximeter and the handheld pulse oximeter. In South Sudan the feature phone was considered expensive, whereas in other countries CHWs and national stakeholders described these phones as being widely possessed:

In our country there are about 30,000 HEW [health extension workers] and about 16,000 health posts. You are not supposed to buy for all of these. It is quite difficult. We have to think of this issue. (Ethiopian national stakeholder)

Furthermore, in South Sudan the risk of theft of high-cost devices such as smartphones was considered high. One national stakeholder raised concerns that thieves would specifically target CHWs because of these devices. Some CHWs were worried about the personal responsibility they would have for repaying the cost if devices were stolen, lost or damaged:

This device can even create insecurity to the CDDs [community drug distributors] when the robbers see you holding it. (South Sudanese national stakeholder)

In Ethiopia, a CHW and national stakeholder both stated that devices performing multiple functions (i.e. measuring the RR and oxygen saturation or heart rate and oxygen saturation) would be considered more cost-effective. A small number of participants in all countries favoured the concept of dual-purpose devices (i.e. a smartphone with pulse oximeter and RR counting capabilities or the additional use of mobile phones for communication purposes), although most did not explicitly mention cost-effectiveness as a reason.

In Cambodia two national stakeholders discussed resource allocation dilemmas. This included the relative benefits of purchasing expensive new diagnostic aids for CHWs versus spending the same amount of money on extra training for CHWs on identifying danger signs in unwell children, purchasing medications or providing patients with transportation to hospital:

What are the causes that our volunteers do not understand the symptoms of pneumonia, both for the newborn and those aged under five? In order to enable the community to know the symptoms, we need to spend money. So, if we have only $100, should we spend the money on the training of how to identify the danger signs or should we spend it on a smartphone? (Cambodian national stakeholder)

## Electricity, batteries and charging

Participants in both groups frequently mentioned the lack of access to electricity in large areas of all four countries. They discussed potential difficulties if aids were highly reliant on electricity for charging. This was widely considered one of the greatest barriers to all the electronic devices being usable or scalable, including mobile phones. Some Cambodian CHWs explained they may be able to access a communal ‘heat store’ or generator to charge devices but this would not be possible in all villages. South Sudanese CHWs stated devices relying on electricity could only be used in larger towns, as more remote villages had no power. Furthermore, Ethiopian CHWs described needing to walk long distances to access power to charge batteries or devices:

As to me, the health post has no electric power access and it is about two to three hours’ walking distance to get electric power. In our case, the electric power access is in one of the schools found in our kebele [municipality – the smallest administrative division]. It has a distance of two to three hours. Therefore, it is difficult to send the phone with students for charging at regular basis. (Ethiopian CHW)

Due to limited electricity access, CHWs and national stakeholders preferred devices with a long battery life. Frequent or daily charging of devices was seen as a barrier to usability and scalability. Many participants liked the long battery life of the ARI timer. However, some CHWs reported difficulties with ARI timers from personal experience. They also mentioned that if electricity access was challenging and/or the battery life was limited, devices would be useless for ongoing assessment of unwell children:

Sometimes it runs out of battery in a week. The battery can be corroded easily. (Cambodian CHW)

Participants gave suggestions on how to use electric devices in remote areas. The idea most frequently mentioned was providing access to solar panels to ensure regular charging:

If we have a small solar panel to charge the battery, it will be much better and much more convenient. We can charge the battery anywhere we want, and we can use the instrument for a long time. (Cambodian CHW)

## Simplicity in the use of diagnostic tools

Participants in both groups stated simplicity was essential to tool usability and scalability. Devices producing automated results, such as pulse oximeters, the RR-counting mobile phone applications and the joint device, were most appreciated by both stakeholders and CHWs. The ARI timer’s inability to produce automated results reduced its perceived usability for some in Cambodia and Ethiopia and other CHWs described it as being confusing to use:

The fingertip pulse oximeter is easy to use because you simply insert a finger and it displays the readings. (Ugandan national stakeholder)

In countries where some CHWs are illiterate or have low education levels, importance was placed on aids being easy to use and ‘convenient for all individuals either educated or not’ (Ethiopian national stakeholder). Most comments on this issue came from national stakeholders and CHWs in South Sudan. Attributes of aids considered important for these users included: having a minimal number of steps to get results; requiring little or no reading; colour-coded classification of results indicating presence or absence of disease; and requiring no advanced technological knowledge to operate the device:

Because [it] is not simple to operate such a phone by someone who is illiterate and also it have a lot of instructions to follow. (South Sudanese CHW)

For the simple phone, we can see the members in VHSGs [Village Health Support Groups] or VMWs [Village Malaria Workers] are mostly old people. Most of the members in VHSGs only know how to receive a phone call or can only make a call to a certain number. It is difficult for us to train them to use the phone. (Cambodian national stakeholder)

Moreover, CHWs in Ethiopia and Cambodia stated they liked the disease classification system of the feature phone application, which minimised the need to interpret results using separate guidelines. It was noted the other RR counting tools lacked this characteristic. Participants in Ethiopia particularly felt this attribute would make the assessment and referral process simpler.

There was resistance to the use of new technological aids, especially smartphones, by national stakeholders across the four countries. Many felt these devices would be too complicated for CHWs to master, and teaching CHWs to use ‘high tech’ devices would be time-consuming. This concern was less prominent amongst CHWs. Many national stakeholders and CHWs preferred familiar technology, such as the existing ARI timer or simple mobile phones:

I am afraid that it is too modern and complicated so that it will be difficult to use. Our volunteers get used to using simple Nokia [feature] phone because it is useful for them and it is also very convenient to use. (Cambodian national stakeholder)

Other simplifying attributes included clear display of results in a large format for older CHWs with poor eyesight, and instructions and steps written in the local language.

## Accuracy of diagnostic tool results

Participants in both groups highlighted the need for accurate aids to assist in classification of children with pneumonia. CHWs emphasised the need for accuracy when discussing problems with the ARI timer, which they often described as being inaccurate. National stakeholders expressed concerns about the accuracy of tools that required CHWs to count breaths for less than one minute and then produced an automated RR. They felt this would produce inaccurate RRs for children with irregular breathing, or amplify mistakes in the counting process:

The other drawback of the application [feature phone application] is it doesn’t count full one minute, it gives estimated number of breath just after tenth or twentieth breath. This implies that it is not appropriate for irregular breathing pattern. (Ethiopian national stakeholder)

Participants reflected on the inherent inaccuracy of CHWs needing to count children’s breaths with distractions such as children crying, moving or other people talking, leading to the need to repeat counting multiple times. This was most commonly mentioned by CHWs referring to previous experiences using the ARI timer.

Both CHWs and national stakeholders liked the smartphone RR counting application which allows users to validate their count findings by listening to the calculated RR and comparing it with the child’s actual RR. They felt this increased its accuracy and usability.

## Durability and sustainability of diagnostic tools

Both groups of participants raised sustainability concerns, both directly and indirectly, during the FGDs. Some mentioned that devices that were not durable or suited to the environment were inappropriate to scale up, because they would easily break and not be replaced. Environmental hazards that CHWs specifically mentioned included: water exposure; being damaged whilst carried in bags; being dropped; or being broken by children during the assessment. National stakeholders raised fewer concerns about aids being damaged.

National stakeholders preferred aids to be low-maintenance. For some devices, such as the pulse oximeters, Ethiopian CHWs and national stakeholders mentioned the impediment of not being able to buy replacement probes or batteries in local shops. Referring to experiences of previous programs and NGO initiatives, some Ethiopian and Cambodian national stakeholders emphasised aids should be supplied in a sustainable manner, using CHWs’ existing mobile phones where possible, rather than providing new phones that might promote donor dependency:

If they can use the instruments for only one year, then the instruments are not usable anymore, we do not support them with more instruments. Therefore, is what we give them sustainable? (Cambodian national stakeholder)

## Acceptability to parents, CHWs and children

Generally, CHWs placed greater emphasis on the need for future aids to be acceptable to children and parents than did national stakeholders. They reported children would not like noisy devices (such as the ticking ARI timer) or those that involved attaching unfamiliar objects to their bodies (in the case of pulse oximeter probes). Cambodian CHWs expressed the greatest amounts of concern on this issue. They felt children would be most comfortable with familiar objects such as mobile phones. Parental acceptability was also considered. CHWs described that parents would prefer aids where they could read the results themselves and understand their implications. This characteristic was highlighted for the joint device and other pulse oximeter devices:

When I use it to count breathing, the children do not know that I count breathing and they may think that I am using a mobile phone. The children do not know so they are not frightened. (Cambodian CHW)

Acceptability of aids to CHWs themselves was important. CHWs preferred tools that were: small and portable; ‘modern’ but simple to use; able to perform multiple functions; and able to provide automated results. They expressed a preference for devices producing fast results. However, national and regional stakeholders did not highlight the importance of devices producing automated results with the same conviction as CHWs. National stakeholders also acknowledged that device portability would be important to usability.

In South Sudan two participants mentioned that certain devices, such as smartphones, might cause problems beyond CHWs’ work. The concern was that husbands may be jealous of their wives having a new and expensive phone and may take it for their own use, creating tensions at home.

## Discussion

The breadth of different views on pneumonia diagnostic aids transpiring from the research activities can be seen amongst the different participant groups. Allocations made by participants in the pile-sorting activities were based on multiple factors, as demonstrated by the many themes that emerged from the FGDs. CHWs and national and regional stakeholders prioritised different characteristics when rating the potential scalability of aids. National stakeholders placed increased emphasis on the need for cost-effectiveness and sustainability. CHWs acknowledged these same characteristics, but also discussed the importance of aids being acceptable to parents, CHWs and children. Both groups acknowledged the problem of using electronic devices in areas without reliable access to electricity. Regarding usability, participants in both groups generally preferred devices that were accurate, simple to use and produced automated results. CHWs were more open to new technology, as evidenced by their positive perceptions of the usability and scalability of smartphone devices, the feature phone ‘app’ and handheld pulse oximeter. National stakeholders favoured using CHWs’ existing mobile phones or the ARI timer as tools, citing familiarity and acceptability, although the significantly lower cost of these options potentially influenced their reasoning.

The differing focuses of the groups can be understood in light of their various practical experiences and responsibilities. NGO and MoH stakeholders are invested in ensuring that the supply and distribution processes of any new pneumonia diagnostic aids are uncomplicated and inexpensive. Alternatively, CHWs are more concerned with practical issues of using devices with children and parents. Both aspects are crucial to the uptake and use of any future pneumonia diagnostic aids in low-income countries. Considering the heterogeneity between study countries, the consistency of themes across the countries and stakeholder groups is striking. Most major country differences were in relation to the suitability of devices to the specific country context and CHW capabilities, further described in what follows.

The majority of participants in both groups rated the ARI timer highly on usability and scalability in the pile-sorting activity (see Figures 2 and 3). Amongst national stakeholders, 97% placed it in piles 1 or 2 for scalability and 94% for usability. CHWs were similarly receptive (94% placed it in piles 1 or 2 for usability and 81% for scalability). This is surprising considering comments made by CHWs in the FGDs describing the device as confusing to use and inaccurate. Additionally, previous findings from FGDs conducted with CHWs in the same countries found that many CHWs had practical difficulties using the ARI timer, such as losing count of the child’s breaths, the time-consuming nature of counting breaths for one minute and then repeating the counting, and low parental confidence in the ARI timer’s use (unpublished data, Malaria Consortium). Observational studies have demonstrated that CHWs’ RR counting is correct in only 39–71% of cases [6–8]. Yet, CHWs and national stakeholders considered the ARI timer’s familiarity, low technology requirements, portability, simplicity and low cost to be very important.

As an alternative RR counting aid, many CHWs felt the smartphone RR counting app would be usable (98% placed it in pile 1 or 2 for usability). However, national stakeholders were less positive and raised scalability concerns due to cost and technology requirements. There is a need for continued research and development into improved RR counting aids, particularly considering the importance placed on RR counting in current WHO pneumonia diagnosis algorithms. In acknowledgement of this current deficit, UNICEF’s supply division released a target product profile for new Acute Respiratory Infection Diagnostic Aids (ARIDA) in 2014 [10]. The vast majority of the key parameters listed in this product profile correlate with important device characteristics discussed by CHWs and national stakeholders in the FGDs, with the exception of the need for devices to have a high level of safety or that it should be easy to maintain hygiene.

Pulse oximeters, particularly the fingertip pulse oximeter, were well received by CHWs and national stakeholders. The fingertip pulse oximeter was deemed the most usable and scalable in the pile-sorting activity. One hundred per cent of CHWs placed it in pile 1 or 2 for usability and 93% of national stakeholders placed it in pile 1 or 2 for scalability. Its portability and simplicity were especially liked. However, as yet, there is no defined role for pulse oximetry within the iCCM cough/difficult breathing algorithm. Future community use of pulse oximeters may enable CHWs to better identify which children require hospital referral and which can be safely managed in the community, thus improving the quality of case management practices and limiting unnecessary referrals [26]. Although hypoxaemia (SpO_2_ < 90%) is predictive of poor outcomes, less is known about the significance of mildly abnormal SpO_2_ findings (90–94%) to oral antibiotic treatment failure in the community [27]. Research to explore the potential usefulness of joint devices is also required, as initial perceptions amongst study participants were positive. If provisions for pulse oximetry are included in future algorithms, our results indicate the pulse oximeters would be acceptable to CHWs and national stakeholders.

Recent research into the use of mobile health (mHealth) approaches to pneumonia diagnostics and the IMCI algorithm suggests potential benefits for health care workers and patients [28–31]. However, the energy-dependent nature of most new pulse oximeters, RR counting aids and mobile phones needs to be addressed before devices are rolled out for community use. The lack of electricity in rural areas, where CHWs often live and work, was mentioned as a drawback to device usability and scalability in all countries by both national stakeholders and CHWs. Alternative power sources such as solar panels, or use of rechargeable batteries with multiple spare sets, would be essential if devices such as smartphones or pulse oximeters were introduced. Similarly, longevity of battery life between charging episodes is essential in regions where access to power is limited.

Different diagnostic aid attributes were considered important in the various countries. For example in South Sudan, where a large proportion of CHWs have low literacy and numeracy levels, devices that did not require reading or counting would be more important than in a country such as Ethiopia where CHWs have a high level of education. Therefore, in some cases it might be beneficial to choose different aids in various settings, instead of enforcing a ‘one size fits all’ approach. The emphasis should be to ensure all aids are accurate, affordable and acceptable to the communities where they will be used.

For all new diagnostic aids, further field evaluation is necessary to determine the accuracy and acceptability of CHWs’ use of devices on neonates and children in community settings. Additionally, robust assessment is needed to quantify the possible public health impact and cost-effectiveness of scaling up new diagnostic aids. This will not only help to evaluate currently available devices but will provide future guidance and inspiration for designing technology for health workers in low-resource settings. Funding to support improved diagnostic aids will require a concerted multi-partner approach and should be given serious consideration by policy makers. Further exploration of potential public–private partnerships in this field may be warranted.

### Study limitations

The multi-country nature of this study provided methodological benefits and challenges. Despite rigorous attempts to standardise data collection methods in the four countries with global research protocols and question guides, slight discrepancies were inevitable. The moderators in each country conducted FGDs in different styles, possibly affecting responses. There was a lack of consistency in collecting demographic data about the FGD participants themselves, thus it has not been reported in this paper. The joint RR counting/pulse oximetry device was not available for demonstration in the majority of groups, which meant it was not included in the pile-sorting activity. Due to the small number of participants who were able to evaluate this device it is difficult to draw conclusions regarding CHW and stakeholder perspectives. Further research and trials on joint devices are required to accurately inform future decision-making.

The sample size of two or three FGDs per country for national stakeholders and CHWs was a limitation, as full thematic saturation amongst sub-groups of participants was not consistently achieved. National stakeholders came from varied backgrounds and expressed more diverse opinions than CHWs Although considerable efforts were made by the research team to invite all appropriate country and regional stakeholders to participate, it is likely that some relevant stakeholders were missed inadvertently. In retrospect, a snowball sampling method might have deepened the pool of potential participants and could be considered in similar future studies. The absence of CHW FGDs was an obvious limitation in Uganda.

## Conclusions and recommendations

This research highlights a diversity of opinions and factors that contribute to the usability and scalability of pneumonia diagnostic aids. National stakeholders and CHWs have different priorities when assessing devices for possible use. However, simplicity and affordability emerged as key characteristics from both groups. It is important to acknowledge that different settings will have different requirements based on available financing, electricity availability and health worker education. To ensure aids are acceptable and sustainable, the views and opinions of both frontline health worker users and health service implementers and funders must be incorporated into the development, manufacturing and procurement process of current and future diagnostic aids. Further research and evaluation are required on the potential use of pulse oximeters by CHWs in pneumonia diagnosis, but our results indicate that certain devices are acceptable to the majority of CHW users, MoH officials and programme implementers.

## Paper context

Pulse oximeters and new respiratory rate counting devices are proposed as improved tools for the community assessment of childhood pneumonia. Views from two main stakeholder groups – community health workers and national stakeholders – indicate that there is a broad range of perspectives regarding key device characteristics. Variation exists between the two groups’ opinions of device usability and scalability. Affordability, sustainability and simplicity are important attributes for diagnostic tools to be useful and acceptable in community settings.
